# The vaccine coverage and vaccine immunity status and risk factors of non-protective levels of antibodies against vaccines in children with juvenile idiopathic arthritis: cross-sectional Russian tertiary Centre study

**DOI:** 10.1186/s12969-021-00594-2

**Published:** 2021-07-05

**Authors:** Mikhail M. Kostik, Natalia A. Lubimova, Irina V. Fridman, Olga V. Goleva, Susanna M. Kharit

**Affiliations:** 1 Saint-Petersburg State Pediatric Medical University, Lytovskaya 2, Saint-Petersburg, Russia 194100; 2 Almazov National Medical Research Centre, Saint Petersburg, Russian Federation; 3 Pediatric Research and Clinical Center for Infection Diseases, Saint-Petersburg, Russia

**Keywords:** juvenile idiopathic arthritis, measles, mumps, rubella, diphtheria, hepatitis B, vaccines, protective level of antibodies against vaccines

## Abstract

**Background:**

Immunosuppressive drugs, incomplete vaccine coverage, immune system dysregulation might be factors of a low level of anti-vaccine antibodies in JIA patients. The study aimed to evaluate vaccine coverage, post-vaccine immunity, and risk factors of non-protective levels of antibodies against measles, mumps, rubella, hepatitis B, and diphtheria in JIA patients.

**Methods:**

A cross-sectional study included 170 children diagnosed with JIA aged 2 to 17 years who received routine vaccinations against measles, rubella, mumps (MMR), diphtheria, and hepatitis B national vaccine schedule. In all patients, the levels of post-vaccination antibodies (IgG) for measles, rubella, mumps, hepatitis B, and diphtheria were measured with ELISA.

**Results:**

Protective level of antibodies were 50% against hepatitis B, 52% - diphtheria, 58% - measles, 80% - mumps, 98% rubella. MMR’s best coverage had patients with enthesitis-related arthritis-85%, compared to oligoarthritis-70%, polyarthritis-69%, systemic arthritis-63%. Diphtheria coverage was 50, 51, 46, 63%, respectively. Incomplete MMR vaccination had 39% patients, treated with biologics, 22% with methotrexate and 14% with NSAID (*p* = 0.025), and 61, 46, 36% for diphtheria (*p* = 0.021). Incomplete vaccination was a risk factor of non-protective level of antibodies against measles (HR = 2.03 [95%CI: 1.02; 4.0], *p* = 0.042), mumps (HR = 6.25 [95%CI: 2.13; 17.9], *p* = 0.0008) and diphtheria (HR = 2.39 [95%CI: 1.18; 4.85], *p* = 0.016) vaccines, as well as JIA category, biologics, corticosteroids and long-term methotrexate treatment for distinct vaccines. One-third part of JIA patients continued vaccination against MMR and diphtheria without serious adverse events and JIA flare. There were no differences between patients who continued MMR vaccination or denied in the means of JIA category and treatment options. Patients, continued diphtheria vaccination rare received methotrexate (*p* = 0.02), biologics (*p* = 0.004), but had higher levels of anti-diphtheria antibodies (*p* = 0.024) compare who omitted vaccination. Methotrexate (OR = 9.5 [95%CI: 1.004; 90.3]) and biologics (OR = 4.4 [95%CI: 1.6; 12.1]) were predictors of omitted diphtheria revaccination.

**Conclusion:**

Children with JIA may have lower anti-vaccine antibody levels and required routine checks, especially in children with incomplete vaccination, biologics, systemic arthritis, and long-term methotrexate treatment. Revaccination of JIA patients was safe and effective.

## Key messages

- Children with JIA have decreased protective levels of antibodies against vaccines.
- Vaccine coverage in JIA children is lower than in healthy peers.
- Incomplete vaccination, biologics, JIA category, and long-term methotrexate treatment are the main factors of non-protective levels of antibodies against vaccines.

## Introduction

Patients with JIA are at greater risk of infections than healthy children due to their aberrant immunity and the use of immunosuppressive drugs [1]. Infection episodes are the main reason for hospital admissions (e.g., pneumonia or sepsis), as well as missing biologics and methotrexate with subsequent JIA flares, impaired JIA remission, and outcomes [2, 3]. Vaccinations can decrease the number of infection episodes, maintain treatment of the disease, and restrain remission [4]. However, many children with PRD stop vaccinating when a diagnosis of rheumatic disease is established [5]. Moreover, many practising paediatricians and pediatric rheumatologists continue to believe that the immune response of JIA patients is disrupted by immunosuppressive drugs and does not lead to the proper level of seroprotection, or they fear that vaccines may cause a persistent autoimmune response, lead to severe disease or relapse for existing diseases [5, 6]. As a result, we have a rather large cohort of immune-compromised children with incomplete vaccination. People with chronic diseases could have a significant risk of preventable superinfection after or during a COVID-19 infection without specific vaccination. An uncontrolled infection outbreak is a disaster for the healthcare system, for the economy, and for social life [7, 8]. We conducted our study to evaluate vaccine coverage, post-vaccine immunity, and the risk factors of non-protective levels of antibodies against measles, mumps, rubella, hepatitis B, and diphtheria in JIA patients.

## Patients and methods

### Study design and patient selection

A cross-sectional pilot study included data from 170 children diagnosed with juvenile idiopathic arthritis - JIA (55 boys and 115 girls). Study inclusion lasted from 2019 to 2020 years.

*Inclusion criteria:* i) the willingness to take part of patients or parents in the study; ii) age from 2 to 17 years; iii) the diagnosis of JIA based on the ILAR criteria (1997) [9]; iv) routine vaccinations against measles, rubella, mumps (MMR) and diphtheria before JIA onset.

Exclusion criteria: i) missing data about vaccines; ii) incomplete vaccination before JIA due to any reasons; iii) scheduled vaccination against measles, mumps, rubella, diphtheria, hepatitis B less than 6 months before study inclusion; iv) recipients of plasma, intravenous immunoglobulin or other similar sources of antibodies in the last 12 months; v) using any other cytotoxic medications or non-biologic DMARDS, except the methotrexate; vi) psoriatic and undifferentiated arthritis due to few patients. The data about the JIA course and treatment obtained from the patient’s charts. We selected an oligoarticular course (less than five active joints), a polyarticular course (extended oligoarthritis, RF-positive, and RF-negative polyarthritis), systemic arthritis, enthesitis-related arthritis. The following classes of immunosuppressive medications used by the patients during study recruitment were taken into account: corticosteroids, methotrexate, biologics.

### National vaccine schedule

Russian national vaccine schedule supposes diphtheria-tetanus-pertussis vaccination in 3, 4½, 6, and 18 months and further diphtheria-tetanus vaccination in 6–7 and 14 years and MMR vaccine at the age of 1 year and 6 years, and hepatitis B vaccination in 0, 1, 6 months. Depending on the number of scheduled vaccines for subsequent analysis, patients цуre divided into two groups with complete and incomplete vaccination. According to the national vaccine schedule, incomplete vaccination means fewer vaccines or vaccine doses to age.

### Assessment of the levels of antibodies against vaccines

In all patients, the levels of post-vaccination antibodies (IgG) for measles, rubella, mumps, hepatitis B, and diphtheria were measured ELISA during study inclusion. IgG concentrations were determined from calibration curves constructed using Dynex Technologies Inc. software (USA). The protective level of antibodies was established in accordance with the criteria specified in the manufacturer’s instructions: for measles IgG - 0.18 IU/ml (coefficient of variation, CV, 8%; analytical sensitivity 0.07 IU/ml), for antibodies to rubella - 10 IU/ml (8%; 2 IU / ml), for hepatitis B (anti-HBs antibodies) - 10 mIU / ml (8%; 2 mIU/ml), for diphtheria - 0.09 IU / ml (7, 5%; 0.004 IU/ml). The minimal protective level of IgG against mumps was established with a positivity coefficient > 1.0. To detect measles, rubella, mumps, and hepatitis B antibodies, we used the commercial kit created by Vector-Best JSC, Russia, and IBL International GMBH (Germany) for diphtheria antibodies. Information about the scheduled vaccination against MMR, hepatitis B, and diphtheria obtained from the personal vaccine certificates.

### Statistical analysis

Statistical analysis was performed with the software STATISTICA, version 10.0 (StatSoft Inc., USA) and MedCalc (MedCalc Software, Belgium). The sample size was not calculated. All continuous variables were checked by the Kolmogorov-Smirnov test, with no normal distribution identified. The quantitative variables were done with median and percentiles (25; 75) for continuous variables and absolute meanings and percentages for categorical variables. For comparison, the categorical variables Pearson’s χ2 test or the Fisher’s exact test in case of expected frequencies < 5 was used, and comparison of two quantitative variables was carried out using the Mann-Whitney test. Survival analysis in each group, with a non-protective level of antibodies against vaccine as the event of interest, was conducted using the Kaplan-Meier method. The log-rank test compared survival curves. Factors significantly associated with a time when the non-protective level was detected or not then tested in a Cox proportional hazards regression model, calculating the Hazard-ratio (HR). *P*-value  <  0.05 was considered statistically significant.

### Ethics

Written consent has been obtained according to the declaration of Helsinki. The Saint Petersburg State Pediatric Medical University’s local Ethics Committee approved the trial protocol (protocol number 9/2 from 02.09.2019).

## Results

### Demographics and vaccine coverage

The characteristics of patients with JIA included in the study are presented in Table 1. We included patients of different ages and JIA categories in evaluating vaccine coverage. The distribution of the JIA categories in the studied population was similar to the JIA distribution in the department. A high proportion of children received corticosteroids (25%), and nearly half received biologics: etanercept – 44%, adalimumab – 29%, tocilizumab – 20% and abatacept – 7%. Sixteen children (9.4%) received more than one biologic drug, consequently.

**Table 1:** Demographic characteristics of patients with JIA

Parameter	Results (*n* = 170)
Girls, n (%)	115 (67.7)
Onset age, years	6.0 (3.7; 9.0)
Study inclusion age, years, (min-max)	2; 17
Median (25%; 75%)	11.4 (7.6; 14.8)
JIA duration, years	3.8 (1.9; 6.5)
JIA categories, n (%):	
Oligoarthritis	73 (42.9)
Polyarthritis	61 (35.9)
Systemic 5arthritis	16 (9.4)
Enthesitis-related arthritis	20 (11.8)
Treatment, n (%)	
Corticosteroids, n (%)	43 (25.3)
Methotrexate, n (%)	154 (90.6)
Methotrexate duration, years.	2.5 (1.1; 5.3)
Biologics, n (%)	82 (48.2)
Biologic duration, years	1.3 (0.1; 4.2)

*Footnotes: continues variables were presented as median and quartiles (25%; 75%), categorical variables in absolute meanings and percentages, n (%).*

### Incomplete vaccination

The majority of patients had been given a restricted number of vaccines, which can explain why a relatively high proportion of the JIA patients were without non-protective levels of antibodies. In the studied population, 170 (100%e) had received one MMR vaccine, and 95 (55.9%) had received two MMR vaccines. Moreover, 82 (48.3%) had received 1–4 vaccines against diphtheria, and 88 (51.7%) had received 5–6 vaccines.

The protective level of antibodies in the whole studied population of JIA patients ranged from 50% (against hepatitis B) to 98.2% (rubella). Among patients with JIA, 50 (42%) had an incomplete complex of vaccines against MMR and 85 (50%) against diphtheria. All patients received a whole complex of vaccines against hepatitis B. The time between the last vaccination and study recruitment was relatively long and ranged from an average of 6.0 (4.1; 9.6) years for diphtheria to 10.9 (7.1; 14.3) years for hepatitis B. The mean level of antibodies against hepatitis B was 9.3 (0.03; 41.9) mIU/ml. The highest MMR vaccine coverage was in patients with enthesitis-related arthritis (85%), followed by patients with oligoarthritis (70%) and polyarthritis (69%), and the lowest coverage was in patients with systemic arthritis (63%). The highest vaccination coverage against diphtheria was in patients with systemic arthritis (63%); lower coverage was in patients with oligoarthritis (51%), enthesitis-related arthritis (50%), and polyarthritis (46%). Comparing children with complete and incomplete vaccinations showed lower levels of antibodies against mumps and diphtheria in the latter. Thus, incomplete vaccination was associated with lower levels of antibodies against mumps and diphtheria (Table 2).

**Table 2:** Levels of antibodies against vaccines in JIA patients depend on vaccine coverage

Parameters	MMR vaccination for age			p^*^
	Whole group (*n* = 170)	Incomplete (*n* = 50)	Complete (*n* = 120)	
Anti-measels IgG, IU/ml	0.2 (0.04; 0.53)	0.2 (0.0; 0.5)	0.2 (0.09; 0.6)	0.181
Patients with anti-measles protective IgG level, n (%)	98 (57.7)	25 (50)	73 (60.8)	0.193
Anti-mumps IgG, IU/ml	2.7 (1.2; 5.3)	1.9 (0.0; 5.1)	2.9 (1.3; 5.3)	0.101
Patients with anti-mumps protective IgG level	136 (80,0)	35 (70.0)	101 (84.2)	0.035
Anti-rubella IgG, IU/ml	79.1 (43.0; 185.1)	69.9 (36.9; 119.6)	87.3 (45.3; 198.3)	0.173
Patients with anti-rubella protective IgG level, n (%)	168 (98.2)	49 (98.0)	119 (99.2)	0.520
Time since the last MMR vaccination, years	7.3 (5.0; 10.3)	5.5 (4.0; 7.5)	7.6 (4.9; 10.6)	0.156
	Diphtheria vaccination			p^*^
	Whole group (*n* = 170)	Incomplete (*n* = 85)	Complete (*n* = 85)	
Anti-diphtheria IgG, IU/ml	0.12 (0.04; 0.31)	0.07 (0.03; 0.22)	0.2 (0.06; 0.4)	0.001
Patients with anti-diphtheria protective IgG level, n (%)	88 (51,8)	34 (40.0)	54 (63.5)	0.002
Time since last diphtheria vaccination, years	6.0 (4.1; 9.6)	5.4 (4.1; 9.0)	6.1 (4.8; 9.3)	0.468

^*^no data due to complete hepatitis B vaccination

**Fig. 1:**
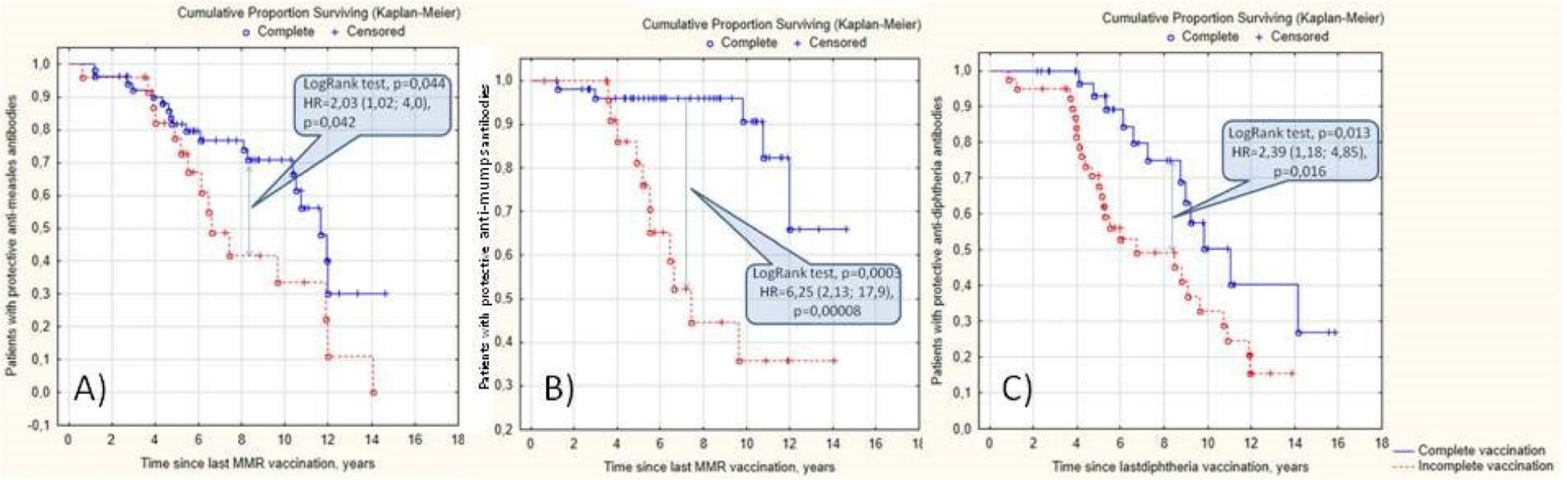
The survival of protective antibody levels against measles (**A**), mumps (**B**), diphtheria (**C**) in JIA patients regarding the completeness of distinct vaccines

Incomplete vaccination is a risk factor for a non-protective level of antibodies against measles (HR = 2.03 [95%CI:1.02; 4.0], p = 0.042), mumps (HR = 6.25 [95%CI:2.13; 17.9], *p* = 0.0008), and diphtheria (HR = 2.39 [95%CI:1.18; 4.85], *p* = 0.016), which was confirmed using Cox proportional regression models (Fig. 1). Incomplete vaccination was strongly associated with the severity of arthritis and the degree of immunosuppression. Incomplete MMR vaccination had 39% of patients treated with biologics, 22% with MTX, 14% with NSAID, and 61, 46, and 36%, respectively, for diphtheria. Positive correlation between biologics and incomplete vaccination against MMR (*r* = 0.2, *p* = 0.008) and diphtheria (*r* = 0.22, *p* = 0.006) was observed. No correlation between incomplete vaccination and methotrexate or corticosteroids was found.

### Risk factors of non-protective levels of antibodies against vaccines

The possible factors that might influence the level of antibodies against the vaccine were the JIA category, treatment modalities, vaccine coverage, and time since the last vaccination, so the best options were time-dependent statistical methods. In survival analysis, we have found differences in patients with protective and non-protective levels of antibodies only against hepatitis B (LogRank test, *p* = 0,018). The lowest probability of having a protective level of antibodies was observed in systemic arthritis compared to oligoarthritis (*p* = 0.008) and polyarthritis (*p* = 0.005).

**Fig. 2:**
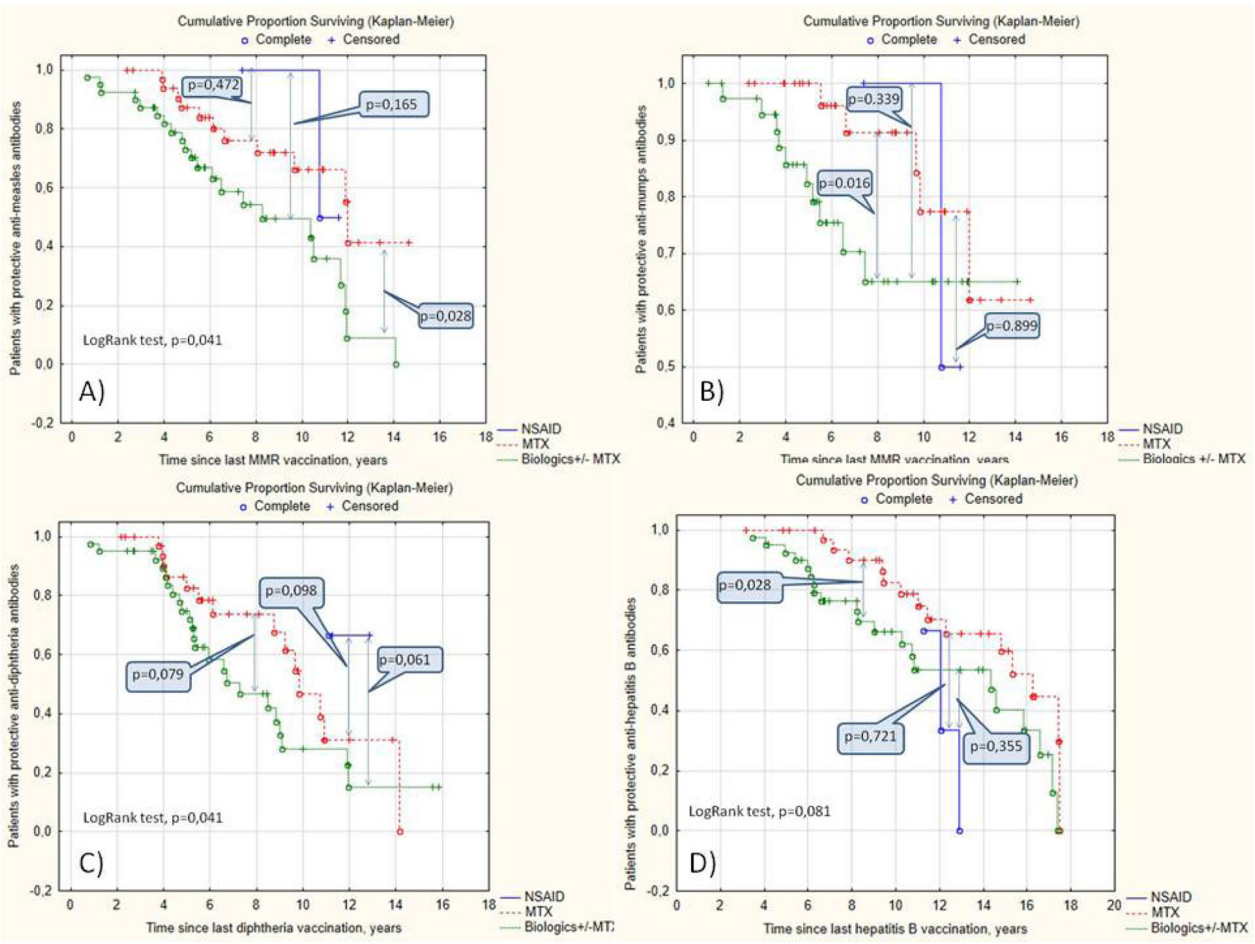
The survival of protective antibody levels against measles (**A**), mumps (**B**), diphtheria (**C**), and hepatitis B (**D**) in JIA patients regarding the treatment options

JIA patients with non-protective levels of antibodies against measles had more extended methotrexate treatment (2.8 [1.3; 6.4] vs. 2.2 [0.9; 3.9] years, *p* < 0.05) and an increased application of the biologics (76% vs. 52%, *p* < 0.05) compared to the patients with protective levels of antibodies. Patients treated with biologics had the lowest probability of having protective antibody levels against measles, mumps, hepatitis B, and diphtheria than MTX and NSAID (Fig. 2).

Patients with non-protective antibodies against mumps had lower vaccine coverage (56% vs. 67%, *p* < 0.05). Patients with non-protective levels of antibodies against diphtheria had lower vaccine coverage as well (38% vs. 61%, *p* < 0.01) and longer duration of methotrexate (3.3 [1.4; 6.7] vs. 1.8 [1.0; 2.9] years, p < 0.01) and biologic treatment (3.1 [1.1; 5.4] vs. 0.9 [0.0; 1.9] years, *p* < 0.05) compared to patients with protective levels. The main risk factors to have non-protective levels of antibodies against specific vaccines are in Table 3.

**Table 3:** Risk factors associated with non-protective levels of antibodies against measles, mumps, rubella, diphtheria, and hepatitis B vaccines (proportional hazard Cox regression models)

Parameters	Measles		Mumps		Rubella		Diphtheria		Hepatitis B	
	HR (95%CI)	р	HR (95%CI)	р	HR (95%CI)	р	HR (95%CI)	р	HR (95%CI)	р
soJIA, yes	1.84 (0.84; 4.03)	0.128	1.43 (0.53; 3.95)	0.492	0.99 (0.05; 18.6)	0.995	2.04 (0.91;4.59)	0.08	2.52 (1.27; 5.0)	0.008
GCS, yes	1.54 (0.91; 2.61)	0.104	0.31 (0.45;1.84)	0.799	0.736 (0.11; 4.88)	0.736	1.89 (1.1; 3.24)	0.02	1.34 (0.77; 2.32)	0.295
МТХ, yes	0.86 (0.39; 1.88)	0.703	1.55 (0.49; 4.88)	0.453	1.53 (0.08; 28.64)	0.776	2.02 (0.71; 5.76)	0.187	0.6 (0.31; 1.15)	0.122
Biologics, yes	2.02 (1.22; 3.32)	0.006	1.76 (0.98; 3.15)	0.057	2.26 (0.5; 9.87)	0.293	1.67 (0.99; 2.8)	0.053	1.2 (0.75; 1.92)	0.453
> 1 biologics, consequent, yes	1.57 (1.13; 2.2)	0.007	1.4 (0.93; 2.09)	0.104	1.82 (0.71; 4.7)	0.213	1.4 (0.98; 2.0)	0.062	1.11 (0.78; 1.58)	0.572
Incomplete vaccination, yes	2.02 (1.02; 4.0)	0.042	6.25 (2.13; 17.9)	0.00008	na^*^	na^*^	2.39 (1.18; 4.85)	0.016	na^*^	na^*^

Footnotes: *CI* confidence interval, *GCS* glucocorticosteroids, *HR* hazard ratio, *MTX* methotrexate, *na* not applicable, *soJIA* systemic onset of juvenile idiopathic arthritis. ^*^ Data was not calculated due to a small number of patients with a non-protective level of antibodies against rubella and no patients with incomplete vaccination against hepatitis B.

### Vaccination during JIA

According to the national vaccine schedule between JIA onset and study inclusion, 58 patients required MMR scheduled revaccination and 76 diphtheria revaccination. Nineteen patients (32.8%) received MMR revaccination and 25 (32.9%) diphtheria, but others omitted vaccination. All patients were vaccinated during the remission without any serious adverse events or JIA flares. Patients who continued vaccination had the only higher age of inclusion in the study at 11.6 (10.7; 15.7) years vs 8.9 (7.0; 11.9) years (*p* = 0.007) and longer JIA duration of 6.9 years (6.3; 11.6) vs 5.5 (3.2; 8.8) years (*p* = 0.03), but no differences in anti-vaccine antibody levels were detected. No predictors (e.g., JIA course and treatment) influencing whether patients were revaccinated against MMR were identified. No differences in the levels of antibodies against measles, mumps, rubella, or the number of patients with protective titers of these antibodies, according to revaccination were found. Patients who continued diphtheria revaccination rarely received methotrexate (84% vs 98%, *p* = 0.02) and biologics (40% vs 60%, *p* = 0.004), but had higher levels of anti-diphtheria antibodies (*p* = 0.024) and a higher proportion of the patients had protective levels of antibodies against diphtheria (60% vs 35.3%, *p* = 0.041). Methotrexate (OR = 9.5 [95%CI: 1.004; 90.3]) and biologics (OR = 4.4 [95%CI: 1.6; 12.1]) were predictors of omitted diphtheria revaccination.

## Discussion

Our study aimed to describe vaccine coverage status and vaccine immunity status in JIA patients in the Russian tertiary centre. The launch of vaccination drastically decreased infections around the world and prevented many deaths [10]. The European Alliance of Associations for Rheumatology recommended the national guidelines for PRD vaccination, but many adult patients with autoimmune inflammatory rheumatic diseases still have an increased risk of vaccine-preventable infections [11, 12]. It is necessary to have a very high proportion (> 95%) of the population have adequate protective antibody levels to achieve herd immunity and block the virus’s circulation for measles, mumps, and rubella [13].

### Vaccine immunity against MMR

According to the official statistics in Saint Petersburg, 97.4–99.6% of the population of varying ages has received the MMR vaccination, but the number of people without protective antibodies in 2019 is higher and ranged from 6.3 to 17.9% in children and 5 to27.1% in adults (18–49 years) [14]. In our study, only 57.7% of JIA patients had protective antibodies against measles [15]. Non-protective levels of antibodies against measles found in 47 patients with completed MMR vaccinations were associated with a higher frequency of systemic corticosteroids (34% vs. 16%, *p* = 0.026), biologics (53% vs. 34%, *p* = 0.040), and proportion of JIA patients who have had the disease more than 3 years (63% vs. 41%, *p* = 0.019). In the whole group, incomplete vaccination, treatment with corticosteroids, biologics, and longer JIA duration affected the antibodies’ level against measles (Figs. 1 and 2, Table 3). The absence of serious adverse events and JIA flare in our study and literature strongly encourages the routine check of antibody levels in patients with risk factors of non-protective titer and recommends an additional vaccination in 12–14 years for low titer [16, 17].

Eighty per cent of our JIA children had protective antibody levels against mumps, which is lower than local epidemiological data [14]. The proportion of rubella seronegative subjects in the population of Saint Petersburg in 2019 ranged from 2.1 to 4.8% in children and 3.0 to 8.2% in adults (18–49 years) and was similar in JIA patients (1.8%) [14]. High protective levels of antibodies against rubella could be measured after 20 years, even after one vaccine dose [15]. Vaccination against MMR in our cohort was safe and effectively similar to children on immunosuppressive therapies from literature (e.g., methotrexate and biologics) [16, 17].

In 400 JIA patients, lower level of antibodies against mumps (OR = 0.4; 95% CI 0.3 to 0.6) and rubella (OR = 0.4; 95CI: 0.3 to 0.7) were detected but not against measles (OR = 1.4; 95%CI: 0.8 to 2.5) compared to 2176 healthy controls with follow-up time lasting 12 months [18]. The lowest levels of antibodies against measles (*p* = 0.025), mumps (*p* = 0.018), and rubella (*p* = 0.077) were detected in cases of systemic JIA. The seroprotection rate among JIA patients was 93.9% for measles, 85.1% for mumps, and 89.8% for rubella, compared to healthy controls: 87.4, 85.0 and 90.3%, respectively. Glucocorticoids and methotrexate did not significantly affect the antibody levels.

In our cohort, incomplete vaccination and treatment with biologics were the main predictors of the nonprotective level against measles and mumps. Biologics affected the anti-measles antibodies’ primarily protective level, while incomplete vaccination predominantly disturbed the anti-mumps antibodies’ protective level (Figs. 1 and 2, Table 3).

### Vaccine immunity against diphtheria

In Saint Petersburg in 2019, the protective antibodies against diphtheria were present in 96.9% of healthy children and 94.1–94.9% of adults, according to the official data [14]. The protective level of antibodies against diphtheria was detected in 51.8% of our JIA patients, which was lower compared to the local population data. In 400 JIA patients, the level of antibodies against diphtheria and tetanus was lower compared to 2176 healthy controls [18]. In 26/29 (89.7%) of patients (2–5 years) with polyarticular JIA who received subcutaneous abatacept, the protective levels of antibodies against diphtheria were detected. Methotrexate and low-dose corticosteroids did not affect the antibody level in both studies [18, 19]. In our study, incomplete vaccination, duration of methotrexate, and biologics applied affected the level of antibodies against diphtheria. Booster revaccinations against diphtheria increased the proportions of subjects with seroprotection in our JIA patients and healthy population and were safe for JIA patients [18, 20].

### Vaccine immunity against hepatitis B

The protective level of anti-HBs antibodies had 50% of our studied JIA group, and all patients had complete vaccination due to the earliest course (before 6 months). In our group, the main predictors affecting antibodies against hepatitis B were a systemic-onset category of JIA and biologics treatment. In the Polish study, 60.7% of JIA patients with similar ages had protective antibody levels against hepatitis B. Girls and patients with polyarticular JIA had the lowest antibody levels [21]. Only half of the patients with different PRD receiving immunosuppressive treatment had a protective anti-HBs level compared to controls – 4% [21]. According to Maritsi D. et al., in 89 patients with a different JIA form, only 55% had a protective level of anti-HBs antibodies and 92% in healthy controls [22].

The vaccine against hepatitis B (HB) is recombinant and may be recommended for vaccination to all immune-compromised children because of safety and efficacy [23–25]. The Japanese College of Rheumatology and the Japanese College of Hepatology considered anti-HB vaccination for unimmunised patients with JIA as soon as JIA has been under control for 3 months [24, 25].

### Vaccine coverage

Many patients (near 40%) who developed arthritis early (before 6–7 years) often miss other scheduled vaccinations, e.g., measles, mumps, diphtheria, tetanus [26]. Usually, most oligoarthritis patients and half of the RF-negative polyarthritis patients risk incomplete vaccination due to younger onset age [27]. About 42 and 50% of our patients had incomplete MMR and diphtheria vaccination, respectively. The risk factors of incomplete MMR vaccination include onset age of less than 4 years (OR = 12.2 (5.0; 29.9), *p* = 0.0000001), duration of JIA > 3 years (OR = 4.4; (2.0; 9.9) and biologics treatment (OR = 2.5 (1.3; 4.9), *p* = 0.008). Incomplete diphtheria vaccination was related to the onset age of less than 4 years (OR = 1.9 (0.9; 3.8), *p* = 0.08), duration of JIA > 3 years (OR = 3.4; (1.8; 6.5), *p* = 0.0002), and biologic treatment (OR = 2.4 (1.3; 4.4), *p* = 0.006).

In a Slovenian study, 35% of 187 PRD children had incomplete vaccination. Hepatitis B and the second dose of MMR were the most often omitted vaccines, similar to our group [5]. In 200 JIA Canadian children, 48, 32, and 39% of patients at 2.5 years, 10.5 years, and their last clinic visit, respectively, had incomplete vaccination (at least one vaccine from MMR, diphtheria, tetanus, pertussis, meningococcus C, hepatitis B, pneumococcus, Haemophilus influenza type B missed) [6]. Measles was the most often omitted vaccine in 42, 23, and 17% of the patients in the same time points, but not mumps, rubella, and diphtheria/tetanus [6].

Near one-third of 715 German JIA patients had incomplete vaccination, mainly due to physicians’ suggestions. The vaccination coverage in preschool children was similar to healthy matches and lower in adolescents (24–79% for diphtheria and tetanus and 60–75% for MMR) with JIA. More incomplete vaccination cases were among patients with polyarticular and systemic JIA who received immunosuppressive therapy rather than oligoarticular and children without immunosuppression [28]. Our patients with immunosuppression therapy (biologics, methotrexate) had minimal vaccine coverage over patients with NSAID alone (Table 3).

Fear of parents or the recommendation of the physicians were the main reasons for incomplete vaccination in Canada (38%) and Brazil (43.5%), similar to our study [29, 30]. There are many concerns from patients, their parents, and healthcare providers about vaccinations’ safety and efficacy in immunocompromised children, for example, JIA [9, 10, 31]. Many physicians had concerns and uncertainty about vaccines’ role in the JIA flares, which lead to the vaccine practice’s interruption or discordance. Physicians make some delays in the scheduled vaccination before a certain period (e.g., stabilising the disease or having a more prolonged remission) or neglect some vaccines [5, 32]. Unfortunately, our primary care physicians and some pediatric rheumatologists acted in the same way, despite the vaccines’ high safety profiles to JIA flares [16, 17]. Usually, vaccine coverage negatively correlated with the patient’s age. Older children have more omitted vaccines [5].

### Vaccine safety and efficacy

Children with PRD may have reduced levels of anti-vaccine antibodies against measles, mumps, rubella, diphtheria, tetanus, and hepatitis B compared to age-matched healthy controls due to the effect of anti-rheumatic drugs on B-cells and memory B-cells [19, 25]. Several studies in PRD patients showed a similar response to healthy controls, but sometimes, the antibody levels might be lower [33, 34]. Several studies showed a contradictory effect of immunosuppressive medications on antibody production and maintenance against measles, mumps, rubella, diphtheria, and tetanus. Thus, these articles’ main benefit is a confirmation of vaccine safety in JIA and PRD patients [16, 17, 19, 20, 22–24, 35].

Revaccination of JIA patients is effective and safe and should be encouraged, especially in patients with incomplete vaccination or low anti-vaccine antibody levels. In the randomized study, the level of antibodies against measles was higher in the JIA patients, who received revaccination, compared to JIA patients who omitted revaccination. No cases of measles, mumps, and rubella were detected after the revaccination [17]. In Australia, the flare rate during 90 days after vaccination was lower than patients’ baseline risk (RR = 0.59 (95% CI 0.39–0.89, *p* = 0.012). The authors explained the reduced risk by the fact that “children may be healthier than usual” by the time of vaccination; a vaccine was delayed before the time “free of viral infection,” and so on [31]. No increased flare risk related to vaccination against influenza, MMR, varicella, human papillomavirus, and hepatitis B in JIA patients was identified [17, 31, 36]. We recommend administering booster MMR vaccinations to patients with a non-protective level of anti-measles antibodies if they are at least 1 year in remission. According to EULAR recommendations and published data about vaccine status, we can strongly recommend routinely checking patient’s vaccine schedule, and in patients with incomplete vaccination and patients having a risk of non-protective levels of antibodies, the physician should routinely check the anti-vaccine antibody levels and encourage patients and their families to continue vaccination with an individual vaccination program [11, 37, 38]. We suppose that a realistic time interval for antibody assessment in a cost-effective manner is 3 years. It is necessary to provide simple algorithms to primary care physicians regarding the management of vaccinations and routine checking of antibodies [36, 39]. Our study’s results found that the data related to vaccination safety and efficacy strongly support the need for missed vaccinations, especially the MMR booster. Interdisciplinary communication between rheumatologists, immunologists, primary care physicians, and healthcare providers is still required to improve JIA patients’ vaccine coverage.

### Limitations of the study

The present study’s main limitations are related to JIA patients’ differences in age, JIA categories, treatment approaches, and the time gap between the last vaccination and study recruitment. The differences in onset ages and the duration of the disease before obtaining the samples were additional factors that influenced antibodies’ survival in the pathogenesis and treatment of diseases. A relatively small sample size leads to the borderline significance of subgroup analysis.

## Conclusion

Children with JIA have lower antibody levels, and many JIA patients have non-protective levels of antibodies and require a routine check. Incomplete vaccination, JIA categories, biologics, corticosteroids, and long-term methotrexate treatment can be supposed to be the risk factors of aberrant vaccine immunity. Individual vaccination schedules are required for JIA patients without protective antibody levels and should be tailored individually with antibody level sampling. It is necessary to decrease the level of apprehension among Russian parents and healthcare providers regarding vaccinations. Further studies on the safety and efficacy of vaccinations in JIA patients are required.

## Data Availability

The datasets used and/or analyzed during the current study are available from the corresponding author on reasonable request.

## Abbreviations

CI: Confidence interval
EULAR: European alliance of associations for rheumatology
HB: Hepatitis B
HR: Hazard ratio
JIA: Juvenile idiopathic arthritis
MMR: Measles, mumps, rubella
OR: Odds ratio
PRD: Pediatric rheumatic disease
RR: Relative risk
TNF- a: Tumor necrosis factor inhibitor

## References

[1] Breda L, Del Torto M, De Sanctis S, Chiarelli F. Biologics in children’s autoimmune disorders: efficacy and safety. Eur J Pediatr 2011;170:15767. 10.1007/s00431-010-1238-z, 2, 15167. 10.1007/s00431-010-1238-z 20556424

[2] Atzeni F, Bendtzen K, Bobbio-Pallavicini F, Conti F, Cutolo M, Montecucco C, Sulli A, Valesini G, Sarzi-Puttini P (2008). Infections and treatment of patients with rheumatic diseases. Clin Exp Rheumatol.

[3] Armaroli G, Klein A, Ganser G, Ruehlmann MJ, Dressler F, Hospach A, Minden K, Trauzeddel R, Foeldvari I, Kuemmerle-Deschner J, Weller-Heinemann F, Urban A, Horneff G (2020). Long-term safety and effectiveness of etanercept in JIA: an 18-year experience from the BiKeR registry. Arthritis Res Ther.

[4] Friedman MA, Winthrop KL (2017). Vaccines and disease-modifying anti-rheumatic drugs: practical implications for the rheumatologist. Rheum Dis Clin N Am.

[5] Bizjak M, Blazina Š, Zajc Avramovič M, Markelj G, Avčin T, Toplak N (2020). Vaccination coverage in children with rheumatic diseases. Clin Exp Rheumatol.

[6] Morin MP, Quach C, Fortin E, Chédeville G. Vaccination coverage in children with juvenile idiopathic arthritis followed at a paediatric tertiary care Centre. Rheumatology (Oxford) 2012;51(11):2046–2050. 10.1093/rheumatology/kes175. PMID: 22864995. 10.1093/rheumatology/kes175 22864995

[7] Felten R, Dubois M, Ugarte-Gil MF, et al. Vaccination against COVID-19: Expectations and concerns of patients with autoimmune and rheumatic diseases [published online ahead of print, 2021 Feb 22. Lancet Rheumatol. 2021. 10.1016/S2665-9913(21)00039-4. 10.1016/S2665-9913(21)00039-4 PMC7906671 33655219

[8] Ellwanger JH, Veiga ABG, Kaminski VL, Valverde-Villegas JM, Freitas AWQ, Chies JAB (2021). Control and prevention of infectious diseases from a One Health perspective. Genet Mol Biol.

[9] Petty RE, Southwood TR, Manners P, Baum J, Glass DN, Goldenberg J, He X, Maldonado-Cocco J, Orozco-Alcala J, Prieur AM, Suarez-Almazor ME, Woo P, International League of Associations for Rheumatology (2004). International league of associations for rheumatology. International league of associations for rheumatology classification of juvenile idiopathic arthritis: second revision, Edmonton, 2001. J Rheumatol.

[10] Crawford N. W., Buttery J. P. Adverse events following immunizations: fact and fiction. Paediatr Child Health 2013;23(3):121–124. 10.1016/j.paed.2012.06.004.

[11] Heijstek MW, Ott de Bruin LM, Bijl M, Borrow R, van der Klis F, Koné-Paut I, et al. EULAR recommendations for vaccination in paediatric patients with rheumatic diseases. Ann Rheum Dis 2011;70(10):1704–1712. 10.1136/ard.2011.150193. 10.1136/ard.2011.150193 21813547

[12] Furer V, Rondaan C, Heijstek M, van Assen S, Bijl M, Agmon-Levin N, Breedveld FC, D’Amelio R, Dougados M, Kapetanovic MC, van Laar JM, Ladefoged de Thurah A, Landewé R, Molto A, Müller-Ladner U, Schreiber K, Smolar L, Walker J, Warnatz K, Wulffraat NM, Elkayam O (2019). Incidence and prevalence of vaccine preventable infections in adult patients with autoimmune inflammatory rheumatic diseases (AIIRD): a systemic literature review informing the 2019 update of the EULAR recommendations for vaccination in adult patients with AIIRD. RMD Open.

[13] Peltola H, Heinonen OP, Valle M, Paunio M, Virtanen M, Karanko V, Cantell K (1994). The elimination of indigenous measles, mumps, and rubella from Finland by a 12-year, two-dose vaccination program. N Engl J Med.

[14] http://78.rospotrebnadzor.ru/551/-/asset_publisher/hC6J/content/%D1%81%D0%B0%D0%BC%D0%B0%D1%8F-%D0%B7%D0%B0%D1%80%D0%B0%D0%B7%D0%BD%D0%B0%D1%8F%3A-%D0%BA%D0%BE%D1%80%D1%8C?redirect=http%3A%2F%2F78.rospotrebnadzor.ru%2F551%3Fp_p_id%3D101_INSTANCE_hC6J%26p_p_lifecycle%3D0%26p_p_state%3Dnormal%26p_p_mode%3Dview%26p_p_col_id%3Dcolumn-1%26p_p_col_pos%3D1%26p_p_col_count%3D2. Data available on 25 June 2021.

[15] O'Shea S, Woodward S, Best JM, Banatvala JE, Holzel H, Dudgeon JA. Rubella vaccination: persistence of antibodies for 10-21 years. Lancet. 1988;2(8616):909. 10.1016/s0140-6736(88)92512-3. PMID: 2902357. 10.1016/s0140-6736(88)92512-3 2902357

[16] Uziel Y, Moshe V, Onozo B, Kulcsár A, Tróbert-Sipos D, Akikusa JD, et al; PReS working party of vaccination study group. Live attenuated MMR/V booster vaccines in children with rheumatic diseases on immunosuppressive therapy are safe: multicenter, retrospective data collection. Vaccine. 2020;38(9):2198–2201. 10.1016/j.vaccine.2020.01.037. Epub 2020 Jan 24. PMID: 31987692. 10.1016/j.vaccine.2020.01.037 31987692

[17] Heijstek MW, Kamphuis S, Armbrust W, Swart J, Gorter S, de Vries LD, Smits GP, van Gageldonk PG, Berbers GAM, Wulffraat NM Effects of the live attenuated measles-mumps-rubella booster vaccination on disease activity in patients with juvenile idiopathic arthritis: a randomized trial. JAMA. 2013;309(23):2449–2456. 10.1001/jama.2013.6768. PMID: 23780457. 10.1001/jama.2013.6768 23780457

[18] Heijstek MW, van Gageldonk PG, Berbers GA, Wulffraat NM. Differences in persistence of measles, mumps, rubella, diphtheria and tetanus antibodies between children with rheumatic disease and healthy controls: a retrospective cross-sectional study. Ann Rheum Dis 2012;71(6):948–954. 10.1136/annrheumdis-2011-200637. 10.1136/annrheumdis-2011-200637 22172491

[19] Brunner HI, Tzaribachev N, Cornejo GV, Joos R, Gervais E, Cimaz R, et al. Maintenance of antibody response to diphtheria/tetanus vaccine in patients aged 2–5 years with polyarticular-course juvenile idiopathic arthritis receiving subcutaneous abatacept. Pediatr Rheumatol Online J. 2020;18(1):19. 10.1186/s12969-020-0410-x 10.1186/s12969-020-0410-x PMC7036185 32087715

[20] Wanlapakorn N, Maertens K, Thongmee T, Srimuan D, Thatsanathorn T, Van Damme P, Leuridan E, Poovorawan Y (2020). Levels of antibodies specific to diphtheria toxoid, tetanus toxoid, and Haemophilus influenzae type b in healthy children born to Tdap-vaccinated mothers. Vaccine..

[21] Szczygielska I, Hernik E, Kwiatkowska M, Rutkowska-Sak L, Kołodziejczyk B, Gazda A (2015). Ocena stężenia poszczepiennych przeciwciał anty-HBs u dzieci z zapalnymi układowymi chorobami tkanki łącznej leczonych immunosupresyjnie. [Assessment of the level of vaccine-induced anti-HBs antibodies in children with inflammatory systemic connective tissue diseases treated with immunosuppression]. Reumatologia..

[22] Maritsi D, Vartzelis G, Soldatou A, Garoufi A, Spyridis N (2013). Markedly decreased antibody titers against hepatitis B in previously immunized children presenting with juvenile idiopathic arthritis. Clin Exp Rheumatol.

[23] Kasapçopur O, Cullu F, Kamburoğlu-Goksel A, Cam H, Akdenizli E, Calýkan S, et al. Hepatitis B vaccination in children with juvenile idiopathic arthritis. Ann Rheum Dis. 2004;63(9):1128–1130. 10.1136/ard.2003.013201. PMID: 15308522; PMCID: PMC1755134. 10.1136/ard.2003.013201 PMC1755134 15308522

[24] Nerome Y, Akaike H, Nonaka Y, Takezaki T, Kubota T, Yamato T, Yamasaki Y, Imanaka H, Kawano Y, Takei S The safety and effectiveness of HBV vaccination in patients with juvenile idiopathic arthritis controlled by treatment. Mod Rheumatol 2016;26(3):368–371. 10.3109/14397595.2015.1085608. 10.3109/14397595.2015.1085608 26471922

[25] Kobayashi I, Mori M, Yamaguchi K, Ito S, Iwata N, Masunaga K, Shimojo N, Ariga T, Okada K, Takei S Pediatric rheumatology Association of Japan recommendation for vaccination in pediatric rheumatic diseases. Mod Rheumatol 2015;25(3):335–343. 10.3109/14397595.2014.969916. PMID: 25381726. 10.3109/14397595.2014.969916 25381726

[26] Berthold E, Månsson B, Kahn R. Outcome in juvenile idiopathic arthritis: a population-based study from Sweden. Arthritis Res Ther. 2019;21(1):218. 10.1186/s13075-019-1994-8. PMID: 31661011; PMCID: PMC6816211. 10.1186/s13075-019-1994-8 PMC6816211 31661011

[27] Sansonetti PJ. Measles 2018: a tale of two anniversaries. EMBO Mol Med. 2018;10(5):e9176. 10.15252/emmm.201809176. PMID: 29685959; PMCID: PMC5938618. 10.15252/emmm.201809176 PMC5938618 29685959

[28] Minden K, Niewerth M, Borte M, Singendonk W, Haas JP (2007). Immunization in children and adolescents with rheumatic diseases [Impfungen bei rheumatischen Erkrankungen des Kindes- und Jugendalters]. Z Rheumatol.

[29] Vazhappilly S, Vanderkooi O, Benseler S, Gerschman T, Johnson N, Luca N, et al.: Immunization status and barriers in childhood rheumatic diseases (abstract). American College of Rheumatology/ Association of Rheumatology Health Professionals Annual Meeting, 2014.

[30] Lima Melo JM, Pileggi GC, Martins De Carvalho L, Leme Ferriani VP: immunization status of children with rheumatic diseases: can the pediatric rheumatologist help to improve? (abstract) pediatric rheumatology European society congress, 2010.

[31] Alfayadh NM, Gowdie PJ, Akikusa JD, Easton ML, Buttery JP. Vaccinations do not increase arthritis flares in juvenile idiopathic arthritis: a study of the relationship between routine childhood vaccinations on the Australian immunization schedule and arthritis activity in children with juvenile idiopathic arthritis. Int J Rheumatol 2020;2020:1078914. 10.1155/2020/1078914, 1078917. 10.1155/2020/1078914 PMC7424527 32831849

[32] Silva C. A. A., Terreri M. T. R. A., Aikawa N. E., Jozélio F. Carvalho III; Gecilmara C.S, Pileggi I V, Virginia P.L. et al. Prática de vacinação em crianças com doenças reumáticas. Rev Bras Rheumatol 2010;50(4):351–355. 10.1590/S0482-50042010000400002.

[33] Heijstek MW, Scherpenisse M, Groot N, Wulffraat NM, Van Der Klis FR. Immunogenicity of the bivalent human papillomavirus vaccine in adolescents with juvenile systemic lupus erythematosus or juvenile dermatomyositis. J Rheumatol 2013;40(9):1626–1627. 10.3899/jrheum.130246. 10.3899/jrheum.130246 23997002

[34] Wiesik-Szewczyk E, Romanowska M, Mielnik P, Chwalińska-Sadowska H, Brydak LB, Olesińska M, et al. Anti-influenza vaccination in systemic lupus erythematosus patients: an analysis of specific humoral response and vaccination safety. Clin Rheumatol 2010;29:605–613. https://doi.org/10.1007/s10067-010-1373-y, 6. 10.1007/s10067-010-1373-y 20140692

[35] Stoof SP, Heijstek MW, Sijssens KM, van der Klis F, Sanders EA, Teunis PF, et al. Kinetics of the long-term antibody response after meningococcal C vaccination in patients with juvenile idiopathic arthritis: a retrospective cohort study. Ann Rheum Dis 2014;73(4):728–734. 10.1136/annrheumdis-2012-202561. 10.1136/annrheumdis-2012-202561 23505231

[36] Toplak, N., Uziel, Y. Vaccination for children on biologics. Curr Rheumatol Rep2020;22(7):26. https://doi.org/10.1007/s11926-020-00905-8. 10.1007/s11926-020-00905-8 32436130

[37] Furer V, Rondaan C, Heijstek MW, Agmon-Levin N, van Assen S, Bijl M, Breedveld FC, D'Amelio R, Dougados M, Kapetanovic MC, van Laar JM, de Thurah A, Landewé RB, Molto A, Müller-Ladner U, Schreiber K, Smolar L, Walker J, Warnatz K, Wulffraat NM, Elkayam O (2020). 2019 update of EULAR recommendations for vaccination in adult patients with autoimmune inflammatory rheumatic diseases. Ann Rheum Dis.

[38] Rondaan C, Furer V, Heijstek MW, Agmon-Levin N, Bijl M, Breedveld FC, D’Amelio R, Dougados M, Kapetanovic MC, van Laar JM, Ladefoged de Thurah A, Landewé R, Molto A, Müller-Ladner U, Schreiber K, Smolar L, Walker J, Warnatz K, Wulffraat NM, van Assen S, Elkayam O (2019). Efficacy, immunogenicity and safety of vaccination in adult patients with autoimmune inflammatory rheumatic diseases: a systematic literature review for the 2019 update of EULAR recommendations. RMD Open.

[39] Bednarek A, Klepacz R (2020). Vaccinology education of nurses and the current immunoprophylaxis recommendations for children with juvenile idiopathic arthritis. J Clin Med.

